# The Prognostic Significance of Histological Subtypes in Patients with Muscle-Invasive Bladder Cancer: An Overview of the Current Literature

**DOI:** 10.3390/jcm13154349

**Published:** 2024-07-25

**Authors:** Francesco Claps, Arianna Biasatti, Luca Di Gianfrancesco, Luca Ongaro, Gianluca Giannarini, Nicola Pavan, Antonio Amodeo, Alchiede Simonato, Alessandro Crestani, Alessia Cimadamore, Rodolfo Hurle, Laura S. Mertens, Bas W. G. van Rhijn, Angelo Porreca

**Affiliations:** 1 Department of Surgical Oncology (Urology), Netherlands Cancer Institute—Antoni van Leeuwenhoek Hospital, 1006 BE Amsterdam, The Netherlands; ls.mertens@gmail.com (L.S.M.); basvanrhijn@hotmail.com (B.W.G.v.R.); 2 Department of Urology, Leiden University Medical Center, 2333 ZA Leiden, The Netherlands; 3 Urological Clinic, Department of Medicine, Surgery and Health Sciences, University of Trieste, 34127 Trieste, Italy; arianna.biasatti@gmail.com; 4 Department of Oncological Urology, Veneto Institute of Oncology (IOV) IRCCS, 35128 Padua, Italy; luca.digianfrancesco@iov.veneto.it (L.D.G.); antonio.amodeo@iov.veneto.it (A.A.); angelo.porreca@iov.veneto.it (A.P.); 5 Department of Urology, Royal Free London NHS Foundation Trust, London NW3 2QG, UK; ongarluc@gmail.com; 6 Urology Unit, Santa Maria della Misericordia University Hospital, 33100 Udine, Italy; g.giannarini@gmail.com (G.G.); alessandro.crest@gmail.com (A.C.); 7 Urology Clinic, Department of Precision Medicine in Medical, Surgical and Critical Care, University of Palermo, 90127 Palermo, Italy; nicpavan@gmail.com (N.P.); alchiede.simonato@unipa.it (A.S.); 8 Institute of Pathological Anatomy, Department of Medicine, University of Udine, 33100 Udine, Italy; alessia.cimadamore@uniud.it; 9 Department of Urology, IRCCS Humanitas Research Hospital, 20089 Rozzano, Italy; rodolfo.hurle@humanitas.it

**Keywords:** bladder cancer, cystectomy, combined modality therapy, variant histology, treatment outcome

## Abstract

Bladder cancer (BC) is the tenth most commonly diagnosed malignancy worldwide. In approximately 25% of cases, it presents as a muscle-invasive disease, requiring a radical treatment. Traditionally, the mainstay of treatment has been radical cystectomy (RC), but in the last decade, bladder-sparing treatments have been gaining growing interest. In particular, trimodal therapy (TMT) seems to yield survival results comparable to RC with less morbidity and better quality of life (QoL) outcomes. In this scenario, we aimed at shedding light on the role of the histological subtypes (HS) of BC and their prognostic significance in muscle-invasive BC (MIBC), treated either surgically or with TMT. We performed a narrative review to provide an overview of the current literature on this topic. When compared with patients diagnosed with conventional urothelial carcinoma (UC) of the same disease stage, survival did not appear to be significantly worse across the reports. But when sub-analyzed for separate subtype, some appeared to be independently associated with adverse survival outcomes such as the micropapillary, plasmacytoid, small-cell, and sarcomatoid subtypes, whereas others did not. Moreover, the optimal management remains to be defined, also depending on the therapeutic susceptibility of each histology. From this perspective, multi-disciplinary assessment alongside the routine inclusion of such entities in randomized clinical trials appears to be essential.

## 1. Introduction

Bladder cancer (BC) is one of the most common malignancies worldwide. Being the tenth most frequently diagnosed, it represents a significant cause of morbidity and mortality [1]. Non muscle-invasive BC (NMIBC) accounts for approximately 75% of BC cases and represents a heterogeneous group of patients with different risks of recurrence and progression to muscle-invasive BC (MIBC) [2]. MIBC accounts for the remaining 25% of BC cases, with 20% presenting as muscle invasiveness at primary diagnosis. 

The current standard of care for non-metastatic MIBC is radical cystectomy (RC) with pelvic lymph node dissection (PLND), with or without neoadjuvant chemotherapy (NAC). Contemporary 5-year survival rates reach approximately 60% [3]. However, RC is associated with significant morbidity, mortality, and quality of life (QoL) impairment [4,5]. Historically, bladder preservation was an alternative option reserved for patients unfit for surgery due to their age or comorbidities or those unwilling to undergo RC [6]. Nevertheless, bladder-sparing options have been increasingly adopted as a valuable treatment in this setting. Trimodal therapy (TMT), entailing maximal transurethral resection of bladder tumor (TURBT), radiosensitizing chemotherapy, and radiation therapy (RT), is the most investigated and widely used approach [7].

Urothelial carcinoma (UC) has a remarkable propensity for divergent differentiation, and up to 25% cases can show morphological variations [8,9]. They can occur in the form of “divergent differentiation” along other epithelial lineages such as squamous, glandular, trophoblastic, or small-cell/high-grade neuroendocrine differentiation, singly or in combination [10,11,12]. Moreover, several “subtypes” (formerly known as variant histologies [VHs]) of UC have been described with distinctive histological patterns, and to some extent, immunohistochemical features, with evidence suggesting derivation from a urothelial lineage. The fifth edition of the World Health Organization (WHO) classification (2022) recognizes the morphological diversity of UC in 11 subtypes, and the guidelines recommend reporting the percentage of each divergent differentiation and or subtype(s)/histologic variant(s) whenever possible [13]. The histological subtype (HS) has been identified as a key pathological feature, as it carries important diagnostic and prognostic implications, reflecting the biology and clinical behavior of bladder UCs. The goal is to improve the risk stratification of patients affected by UC with the aim to facilitate clinical decision making and treatment tailoring [14,15,16].

Historically, a “treatment intensification” strategy with RC as a cornerstone was advocated in the scenario of VH MIBC, but recent evidence has highlighted that aggressive histology does not predict the superiority of RC over TMT [17].

This review presents an overview of the most recent data regarding the oncological outcomes of patients with MIBC with a HS treated with TMT or RC, with or without NAC and/or adjuvant chemotherapy (AC). The aim of this study is to observe the role of the HSs of BC as prognostic factors when focusing on both surgical and bladder-sparing treatment modalities for MIBC.

## 2. Materials and Methods

A comprehensive database search of the literature produced until May 2024 was performed. Relevant manuscripts were selected from the PubMed, Embase, and Medline archives. Only studies in the English language were included. Reports on the HSs in NMIBC were excluded. Case reports and case series were not considered. Oncological variables were reported when provided by the authors. Overall survival (OS) and/or overall mortality (OM), disease-specific survival (DSS), cancer-specific mortality (CSM), and recurrence-free survival (RFS) were considered as the main outcomes.

## 3. Results

A total of 13 papers met the inclusion criteria and were, therefore, included in the review process. Among the selected reports, five considered TMT, and the remaining eight used a surgical treatment with curative intent. The main outcomes were reported separately, according to the therapeutic treatment received.

### 3.1. Prognostic Significance of MIBC Subtypes in Patients Treated with RC

The main findings of the selected studies reporting the oncological outcomes after RC for MIBC are shown in Table 1.

Martini et al. (2021) [18] described the outcomes of a population of 1894 patients with pure UC and 528 patients with a HS who underwent RC with the prior administration of NAC in 10% of the cases, AC in 17%m and both NAC and AC in 1%. Of the 528 patients with VHs, 9% had non-urothelial variants, 10% had mixed subtypes, 29% had squamous differentiation, and 14% had the micropapillary subtype. For this group, the 5-year OS was 48% and 5-year CSS was 62%. Overall, patients with a HS and the differentiation of UC had a higher risk of recurrence at 10 years, as compared with the pure UC group (RFS 30% vs. 51% at 10 years).

Our study croup [19] considered a cohort of 1082 patients treated with upfront RC, of whom 72.5% had pure UC, while the remaining 27.5% harbored a HS and divergent differentiation. Squamous differentiation (along with UC) was the most common and was present in 15.3% of the patients. The micropapillary subtype was reported in 3.7% of the cases, sarcomatoid in 2.7%, glandular differentiation in 1.7%, 1.3% lymphoepithelioma-like in 1.3%, small-cell in 1.2%, clear-cell in 0.7%, nested in 0.6%, and the plasmacytoid subtype in 0.3%. In the overall cohort, at univariable analysis, the sarcomatoid, plasmacytoid, and small-cell VHs exhibited a worse DSS than pure UC with a HR of 1.61, (95% CI 1.01–2.59) [*p* = 0.047], HR of 6.21, (95% CI 1.99–19.4) [*p* = 0.002], and HR of 1.93, (95% CI 1.09–3.73 [*p* = 0.03]), respectively. Lymphoepithelioma-like VH had a HR of 0.20, (95% CI 0.05–0.82 [*p* = 0.025]). At multivariable analysis and considering the overall cohort, after adjusting for all clinico-pathological predictors, the plasmacytoid and small-cell variants remained independently associated with a worse DSS in the overall cohort: HR pf 3.37 (95% CI 1.06–10.7 [*p* = 0.04]) and HR of 1.93 (95% CI 1.02–3.79 [*p* = 0.04]), respectively.

The series from Naspro et al. [20] analyzed data from a single-center cohort of 525 patients who underwent RC. The histological subtypes were as follows: pure UC at 75%, squamous differentiation at 14.9%, glandular differentiation at 1.5%, nested subtype at 1.5%, micropapillary subtype at 2.9%, small cell at 1.3%, sarcomatoid subtype at 1.7%, and the plasmacytoid subtype, microcystic subtype, and trophoblastic differentiation were considered as “Plasm-Troph-MC”, consisting of 1.2% of the population. Of those, 36% of patients presenting pure UC and 51.1% of patients with VHs experienced a cancer recurrence during the follow-up. At Kaplan–Meier survival analysis, patients with the micropapillary subtype, sarcomatoid subtype, or small-cell differentiation were associated with reduced survival and DSS rates when compared with the other subtypes and pure UC. At multivariable analysis, both MP-Sarc-SC and the other histological variants were an independent predictor of CSM (HR: 2.6 and 1.8, respectively, *p* < 0.005). The sarcomatoid subtype was independently associated with a higher risk of recurrence and CSM, while patients with squamous differentiation, small-cell, and other urothelial subtypes were at a greater risk of dying from their disease. At multivariable analysis, when considering each single VH, none had a significant impact on OS.

Janopaul-Naylor et al. [21], in 2021, evaluated a cohort of 2093 patients who underwent RC for MIBC. In their cohort, subtypes were represented as follows: squamous cell carcinoma (35%), adenocarcinoma (15%), neuroendocrine differentiation (15%), the micropapillary subtype (10%),and other histological subtypes (25%). Overall, the median OS was 19.1 months with a 5-year OS of 33.2%. For squamous cell carcinoma, the median OS and 5-year OS were 14.2 months and 33.8%, and for adenocarcinoma, 31.9 months and 31.2%, respectively. For neuroendocrine differentiation, they were 18.5 months and 33.8%, and for the micropapillary subtype, 22.5 months and 24.1%, respectively. For other variants of UC not otherwise specified, the median OS and 5-year OS were 16.7 months and 33.9%, respectively.

The study group of Moschini et al. [22] presented data from a cohort of 1067 patients who underwent RC for MIBC. Of these, 68.3% had pure UC, whereas the remaining 31.7% harbored a HS. Of the 31.7% patients, 2.0% were diagnosed with the sarcomatoid subtype, 0.9% with the lymphoepitelial subtype, 1.8% with small-cell carcinoma, 10.2% with squamous differentiation, 8.3% with the micropapillary subtype, 2.2% with glandular differentiation, 3.2% with a mixed subtype, and 3.1% with other variants. OS and CSS were considered as the main oncological outcomes in this study. The small-cell variant was independently associated with worse survival outcomes. Moreover, the mixed subtype (defined as the presence of more than one histologic subtype in the same bladder specimen) showed an increased risk of recurrence after RC at univariable analyses. Finally, according to their results, the micropapillary subtype and squamous differentiation were not associated with adverse outcomes after RC.

Monn et al. [24], in 2015, reported the outcomes of squamous differentiation and the micropapillary, sarcomatoid, and plasmacytoid subtypes. A total of 624 patients were included, 74.0% had non-variant histology, 10.9% had squamous differentiation, 4.5% had the micropapillary subtype, 4.0% had the plasmacytoid subtype, 2.4% had the sarcomatoid subtype, and 4.2% had other HSs, including glandular differentiation, nested, lymphoepithelioma-like, and clear cell. All patients underwent RC, and 13% received NAC. The median OS reported was 68 months for squamous differentiation, 24 months for the micropapillary subtype, and 22 months for the plasmacytoid subtype, respectively.

Stroman et al. [23] presented a retrospective analysis of 430 patients that underwent RC. The cohort consisted of pure UC at 83%, squamous diff. at 9.5%, glandular diff. at 3%, micropapillary at 1.8%, plasmacytoid at 0.69%, nested at 0.9%, microcystic at 0.9%, sarcomatoid at 2.0%, and clear cell at 0.2%. The 2-year OS was 85% for pure UC and 71% for all other VHs, whereas the 5-year OS was 70% for pure UC and 47% for all other VHs, respectively.

Fairey et al. [25] retrospectively analyzed a cohort of 1380 patients affected by MIBC, 97.6% of which presented pure UC and 2,4% the micropapillary subtype. All patients received RC and PLND with or without the administration of NAC or AC. The 5-year OS was 59% for pure UC and 67% for the micropapillary subtype. At univariable Cox regression, HS was not associated with OS (HR 0.99, 95% CI 0.60–1.62, *p* = 0.96) or RFS (HR 1.33, 95% CI 0.75–2.36, *p* = 0.33). The 5-year OS was 59% and 67% for pure UC and the micropapillary subtype, respectively. The 5-year RFS was 67% and 58% for pure UC and the micropapillary subtype, respectively.

### 3.2. Prognostic Significance of MIBC Subtypes in Patients Treated with TMT

The main findings of the selected studies describing the oncological outcomes after TMT for MIBC are reported in Table 2.

Janopaul-Naylor et al. [21], in 2021, considered a population of 356 patients who had received TMT for MIBC. Their histology was as follows: squamous cell carcinoma (26%), adenocarcinoma (11%), neuroendocrine differentiation (38%), the micropapillary subtype (5%), and other histological subtypes (21%). Overall, the median OS was 19.0 months with a 5-year OS of 26.4%. For squamous cell carcinoma, the median OS and 5-year OS were 12.6 months and 21.7%, respectively. For adenocarcinoma, they were 13.4 months and 19.3%. For the neuroendocrine subtype, they were 25.2 months and 32.0%, respectively. For the micropapillary subtype, they were 38.5 months and 0%, respectively. For other subtypes of UC not otherwise specified, the median OS and 5-year OS were 23.3 months and 29.9%, respectively.

Krasnow and colleagues in 2016 [26] reported 303 MIBC cases treated with TMT. Pure UC accounted for 78.2% and HSs were 21.8%, of which: 7.9% were squamous diff., 7.6% were glandular diff., 0.7% were squamous and glandular diff., 2.7%were the sarcomatoid subtype, 1% were the micropapillary sub., 1% were neuroendocrine diff., 0.3% were the nested subtype, 0.3% were the nested subtype with glandular diff., and 0.3% were clear cell, respectively. The 5-year OS for the pure UC group was 61% and for HSs it was 52%, whereas the 10-year OS was 42% for both the pure UC and histological subtypes groups. DSS was also considered as a main outcome. The 5-year DSS was 75% for pure UC and 64% for HSs; the 10-year DSS was 67% for pure UC and 64% for HSs, respectively.

In 2018, Van de Kamp et al. [27] presented a series of 65 patients all affected by small-cell MIBC treated with bladder-sparing chemotherapy and radiotherapy after maximal TURBT. The median OS was 52 months, median CSS was 134 months, and median RFS was 22 months. 

In 2020, Nagumo et al. [28] described a population of 148 MIBC patients: 92.6% were affected by pure UC and 7.4% by HSs or divergent differentiations, of which 64% were squamous cell and/or glandular differentiations and 36% were the sarcomatoid subtype, plasmacytoid subtype, signet ring cell, and clear cell. All underwent TMT. The 3-year and 5-year OS were 87% and 84% for pure UC and 88% and 75% for the HS group, respectively. The 3-year and 5-year CSS were 90% and 89% for pure UC and 88% and 75% for the HS group, respectively. The 3-year PFS rates were 84% for pure UC and 81% for the HS group, whereas the 5-years PFS rates were 80% for pure UC and 58% for HSs, respectively.

By using the Surveillance, Epidemiology and End Results (SEER) database (2000–2018), Barletta et al. evaluated patients with a HS receiving TMT. The authors aimed to evaluate four HSs: pure UC, squamous and adenocarcinoma differentiations, and neuroendocrine carcinoma of the bladder. Only cT2N0M0 patients were considered. Using multivariable Cox regression models with CSM as the endpoint of interest, only squamous differentiation had significant worse disease-specific outcomes (HR 1.98, 95% CI 1.5–2.61, *p* < 0.001) and exhibited a lower 3-year CSM than pure UC (35% vs. 57%) [29].

## 4. Discussion

Over the last years, growing attention has been concentrated on bladder-preserving treatments, and in particular, it has been shown that chemoradiotherapy after extensive/maximal TURBT is a viable alternative for patients with MIBC [30,31,32,33]. What comes in addition is avoiding the morbidity and mortality of radical surgery and preserving the bladder with a better QoL and psychological benefits [34,35,36]. Focusing on oncological outcomes, an encouraging recent systematic review and meta-analysis showed no statistically significant difference in terms of the OS between patients undergoing RC or TMT [37].

Controversy exists about the superiority of one treatment over another. In this setting, adjuvant treatments such as AC may contribute to better outcomes. Querying the National Cancer Database (NCDB), Koehne et al. identified 4469 patients with HSs, and squamous differentiation was the most represented entity (31%). Using multivariable models, receiving AC was independently associated with a significantly longer OS for the squamous, sarcomatoid, and micropapillary cohorts [38]. Conversely, considering a similar cohort of 3963 individual patients and their data (IPD), Zamboni et al. found no significant benefit on survival outcomes (all *p* > 0.05) among AC candidates with VHs [39]. As VHs present distinct biological behavior, identifying those patients who may most benefit from RC or bladder-sparing treatment such as TMT represents an important clinical unmet need [19]. Furthermore, it is not only relevant to understand the sensitivity of each variant to a specific treatment, but it is also of paramount importance to fully understand which treatment sequence yields the most effective outcomes in the context of personalized therapy [40]. To clarify this, here, we reviewed the current literature on this topic to provide a state-of-the-art overview in regard to the efficacy of radical treatments, either surgical or bladder-sparing, in this challenging clinical case scenario.

Considering the common practice of the past years, morphologic subtypes of BC were prone to being under-recognized, under-diagnosed, or misclassified for several reasons [41]. Morphologically, tumors are highly heterogeneous, and the recognition of subtypes can be compromised by undersampling. This is reflected by the variable concordance rates between TURBT and RC specimens reported in the literature; whilst some studies demonstrate a relatively poor concordance [9,42], others report rates as high as 83.6% [43]. Intratumoral heterogeneity in RC specimens was described by Warrick et al. [44], observing significant molecular intratumoral heterogeneity between VHs. This shows that different regions of a tumor are genetically distinct, and, therefore, if an area of genomically aggressive tumor is not sampled, a patient could be incorrectly assigned to a lower-risk group. Other reasons for misdiagnoses of VHs include fixation artefacts, high interobserver variability, changing diagnostic criteria, and the lack of supplementary tests to confirm variants’ diagnosis [45]. Altogether, increased awareness, recognition, and improved reporting by pathologists has led to a rise in the incidence of subtypes. So, it is of utmost importance to be aware of their meaning and clinical impact [46,47].

Considering radical surgery for MIBC, according to the results of our analysis, the presence of some (small-cell and plasmacytoid) subtypes or variants is associated with worse survival outcomes as compared to conventional UC. This further confirms the data already reported in the literature. Historically, BC with subtypes of UC or other non-urothelial bladder tumors have worse outcomes and often present with more advanced disease [48]. However, not all studies have agreed on this. Furthermore, comparing the results of all reports is challenging given the heterogeneity of the analyses performed.

Mori et al. [49], in their systematic review on VHs, highlighted that, in many studies, several subtypes have been commonly analyzed together or combined into specific groups. Therefore, assessing the real impact of each individual subtype on oncological outcomes was often impossible. Taking this as a benchmark, a noticeable finding of the studies we reviewed are the results emerging when a sub-analysis of the specific outcomes of each single HS is performed. 

A wide overview with relevant data on the prognostic impact of each subtype was provided by our study group, considering each specific subtype adjusted by different disease stages [19]. In the overall cohort, the plasmacytoid subtype was associated with a worse DSS at univariable analysis, and even after adjusting for all clinico-pathological predictors, it remained independently associated with worse survival outcomes at multivariable analysis. The sarcomatoid subtype showed a worse DSS compared to pure UC at univariable analysis in the overall cohort. It also demonstrated features of epithelial-to-mesenchymal transition, which has been historically associated with aggressive biological behavior [50]. In addition, we found interesting data regarding the clear-cell subtype, which is a very uncommon histology. In this study, it resulted in being independently associated with a worse DSS compared to pure UC in the setting of localized MIBC. Due to the paucity of data on this VH, it could be used to select patients who require an intensive surveillance protocol or adjuvant treatments. On the other hand, the lymphoepithelioma-like subtype exhibited the best survival trend in this analysis. After controlling for standard predictors, the lymphoepithelioma-like subtype resulted in being potentially associated with a better DSS as compared to pure UC.

Naspro et al. [20] reported that histological subtypes are statistically associated with worse survival outcomes and a higher risk of recurrence after RC. Moreover, MP-Sarc-SC (the micropapillary subtype, sarcomatoid subtype, and small cell) emerged as the subgroup with the poorest prognosis among all subtypes. On the other hand, a second-level multivariable analysis stratified for each single variant found that sarcomatoid differentiation statistically impacted CSM and the risk of disease recurrence, while it was weakly associated with OM.

Stroman et al. [23] reported that the presence of subtypes affects the OS and CSS. However, when sub-analyzing for single subtypes, only squamous differentiation (the most common one, followed by glandular differentiation) was associated with worse outcomes after RC in both uni- and multivariable analysis. The presence of other HSs was not associated with a poorer prognosis. Similarly, in the series of Monn et al. [24], the micropapillary subtype and squamous differentiation were found to be associated with worse survival after RC in multivariable analyses. Moschini et al. [22] reported that several histologic subtypes are predictors of a higher recurrence risk, worse CSS, and worse OS after RC in univariable analyses, with the micropapillary subtype and squamous differentiation being the most frequent. Notably, after adjusting for all confounders in multivariable analyses, only small-cell carcinoma retained its effect on survival outcomes.

In regard to the micropapillary subtype, which is one of the most frequently reported, Fairey et al. [25], after adjusting for pT stage, showed that it was not an independent risk factor for worse outcomes after RC with or without perioperative chemotherapy. Conversely, previous reports highlighted the micropapillary subtype as an aggressive variant of UC able to influence the prognosis of RC candidates [24]. Moreover, Martini et al. [18], in their study, underlined that the micropapillary histology caused the greatest risk of disease recurrence after RC compared with other HSs and conventional UC. They considered urothelial subtypes, non-urothelial subtypes, and mixed ones, with the aim to create a personalized follow-up scheme of patients harboring HSs. They also found that patients with the micropapillary subtype presented a shorter median time to recurrence as compared to those with pure UC.

All these results confirm that establishing the real impact of HSs on outcomes and relative disease management represents a clinical challenge. In this context, evidence is often extracted from limited cohorts and is underreported in the literature.

Moving to bladder-sparing treatments, in the last decade, TMT seems to be the mainstay to strive for.

Nagumo et al.’s [28] findings suggest that TMT can be a feasible alternative treatment option for MIBC, even in patients with squamous and/or glandular differentiation. For other subtypes such as the micropapillary, sarcomatoid, and plasmacytoid subtypes, definitive conclusions were not arguable due to the limited sample size. Until sound evidence is produced, special caution should be advised when performing bladder-preserving therapy for such aggressive variants. 

An interesting study in the prospective of the review was that from Janopaul-Naylor et al. [21]. Their results showed that the administration of TMT was associated with a similar overall survival when compared to RC in MIBC with morphologic subtypes, the majority being neuroendocrine and squamous differentiation. It must be acknowledged that, by subgroup analysis, for the micropapillary subtype, the median OS and 5-year OS were 38.5 months and 0% with TMT and 22.5 months and 24.1% with surgery.

The largest study assessing the efficacy of TMT in HSs was that by Krasnow et al. [26]. Most patients presented squamous and glandular differentiation. After TMT, these patients had similar oncological outcomes to their counterparts with pure UC, with no significant difference in terms of OS and DSS. The second most common subtype was the sarcomatoid one. The 5-year DSS was 56% (95% CI: 15–84%) and was not significantly different from that of pure UC 75% (95% CI: 68–81%) and *p* = 0.7). Moreover, in this series, patients with the micropapillary subtype appeared to respond similarly to the patients with pure UC after TMT, but the sample size was too small to be statistically significant. 

An interesting drive for future studies came from Krasnow et al. [26], in particular due to the lack of reports existing on this topic. They demonstrated, in fact, that the response to TMT is also primarily driven by tumor stage. Patient characteristics, tumor stage, the presence of hydronephrosis, and the completeness of the TURBT are the most important clinical factors to be considered when deciding if a patient is an appropriate candidate for TMT. Hence, the presence of a UC subtype should not, per se, represent an absolute contraindication to bladder-sparing therapy. This could be of paramount importance in the management of MIBC presenting histological subtypes.

A key finding of this review is the cross-over between the WHO 2016 definition of VH, the revised definition of HS provided by the 2022 edition, and the term “divergent differentiation”, including UC with squamous differentiation, UC with glandular differentiation, UC with trophoblastic differentiation, and UC with Mullerian differentiation (clear cell adenocarcinoma), which remained consistent across different versions of the “Blue Book”. Therefore, caution is needed when comparing the results of different studies. Moreover, we noticed that, despite the recommendation to report the percentage of divergent differentiation and or HS/VHs when possible, this item was included by only a few studies. Here, querying the SEER Registry, Barletta et al. found worse tumor-specific outcomes among cT2N0M0 patients harboring a squamous differentiation. However, a mixed setting could have biased these findings. Beyond the inherited limitations due to a retrospective registry-based analysis, according to the updated (ed. 2022) WHO Classification of Tumors of the Urinary System, the evaluated entities were mostly considered as divergent differentiations rather than subtypes of invasive UC [51]. However, it must be recognized that this design is a common feature across all evaluated studies. Thus, caution is advised when interpreting these results, as divergent differentiation may exhibit different biological behavior and treatment responses across different lines and therapy regimens [19,23,52].

These data suggest two fundamental assumptions. First, considering that not all subtypes of UC share the same behavior, it might be an oversimplification to consider all morphologic subtypes as a unique group. Each histology needs to be evaluated for its clinicopathological features, natural history, tumor biology, and therapeutic susceptibility. This leads to the second endpoint, which is the key role that the multidisciplinary team plays. Since the “one size fits all” approach cannot represent the optimal strategy in such a heterogeneous setting, a multimodal approach underpinned by the available evidence for each histology can lead to the best treatment, since different histologies might differently benefit from chemotherapy, radiation, and surgery.

This review has several limitations. First, the narrative nature of this review intrinsically creates a bias. All the papers selected were retrospective studies, so selection bias in the populations studied might be present in each of them. Moreover, the heterogeneity of the oncological outcomes taken as reference and collected in the selected studies could represent an issue. Furthermore, the short follow-ups and small numbers of patients harboring specific HSs limit the generalizability of these findings. Some included reports also considered historical cohorts in which different temporal practice patterns may have existed, potentially influencing outcomes.

Another limitation is the update in the WHO histological classification of urothelial tumors of the urinary tract. In fact, in 2022, the fifth edition of the WHO classification replaced the 2016 one, creating a bias in interpreting the data from studies performed before and after changing the reference. In this context, the subdivision of the nested subtype is worth mentioning. The fifth edition of the “Blue Book” recognized the “large nested” as a specific subtype given its unique morphologic and molecular features. Moreover, pathological specimens did not undergo a secondary or central revision in all reports. In most of them, the specimens were evaluated at each institution without a secondary review by a central institution, resulting in potential bias in identifying and reporting subtypes. In addition, many studies did not report the proportion of the histological subtype in the specimens obtained, and this may have affected the survival outcomes. In this sense, Moschini et al. [22] reported that the presence of mixed variants (defined as the presence of more than one histologic subtype in the same bladder specimen) is related to a detrimental effect on survival outcomes after RC. 

Finally, the sample size of non-pure UC was very limited compared to the population with pure UC studied in almost all studies. This is particularly evident when discussing the TMT outcomes. It must be acknowledged that, in the past, clinical practice subtypes often represented a contraindication to bladder-preserving strategies, and these patients were often excluded from clinical trials [25]. However, this also explains the limited confidence that the urological community grants to bladder-sparing strategies in the context of HSs of UC. 

There are a lack of studies addressing the difference in survival outcomes according to different HSs after TMT. This could be the horizon for future clinical research. Trials such as RAIDER [53] and BL13 [54] will provide relevant information, as HSs are allowed among inclusion criteria. Particularly, the NCT03768570 (BL13) is a randomized phase II trial assessing TMT with or without adjuvant durvalumab to treat patients with MIBC. Its results are expected in March 2025 [54]. Furthermore, compelling evidence is also coming from novel immune checkpoint inhibitors (ICIs), with some trials opening the doors to VHs, historically labelled as an exclusion criterion. For instance, the PURE-01 study showed that both the lymphoepithelioma-like and squamous variants were the only VHs sensible to neoadjuvant pembrolizumab immunotherapy [55].

Furthermore, comprehensive molecular analyses of HSs of UC may harbor potential therapeutic implications. Meric-Bernstam and colleagues showed promising results about the efficacy of trastuzumab deruxtecan (T-DXd) among 267 patients harboring unresectable or metastatic solid tumors, including BC (15.4%) with human epidermal growth factor receptor 2 (HER2) overexpression [56]. T-DXd is a HER2-directed antibody–drug conjugate composed of a humanized immunoglobulin G1 anti-HER2 monoclonal antibody, a tetrapeptide-based cleavable linker, and a potent topoisomerase I inhibitor payload [57]. HER2 overexpression and amplification are common in the micropapillary subtype, as demonstrated by Zinnall et al. [58]. Thus, molecular profiling could represent a crucial step towards the stable implementation of targeted therapy in clinical daily practice, also in the context of HSs of UC.

## 5. Future Perspectives

BC develops via two distinct molecular pathways, resulting in NMIBC and MIBC. Common alterations in MIBC include a loss of function of key tumor suppressors, leading to escape from cell cycle checkpoints and the dysregulation of major signaling pathways. TP53, PTEN, and RB1 are frequently mutated, and regulators of their pathways are also altered [59].

BC represents a morphologically and genomically heterogeneous disease with a wide spectrum of morphologic subtypes. Some of these display specific molecular alterations, in particular, in plasmacytoid, micropapillary, and small-cell carcinoma, several corresponding mutations have been identified. 

Tailoring personalized treatment based on the molecular profiling of different types of BC and their consequent subtyping represents a mandatory step for precision medicine in this setting [60]. Several molecular classifications of BC have been reported over the last decade and further summarized in the so-called consensus subtypes. Currently, the consensus on the molecular classification of BC identifies six molecular categories: luminal papillary, luminal non-specified, luminal unstable, stroma rich, basal-squamous, and neuroendocrine-like, each of them with their own molecular signature [59]. Whether or not the molecular subtyping of BC has implications for prognosis and treatment remains controversial [59,60,61]. 

Nowadays, the molecular taxonomy of BC remains in the hypothesis-generating data phase, with still no implication in clinical practice. Further studies are needed to allocate the place of VHs in this context.

## 6. Conclusions

The dogma of a “poor prognosis” among BC patients harboring subtypes of UC needs to be reinterpreted by specifically considering each single entity. It is of clinical importance that rare and complex cases are granted a careful multidisciplinary management given the limited role of currently available data in supporting one treatment modality over another. Regarding clinical research, there is a need for prospective, multi-institutional studies examining the impact of different histologies of MIBC on survival outcomes, especially when bladder-sparing treatments are administered. Given the relative rarity of HSs, the stable implementation of a dedicated uropathologist in multidisciplinary teams and multi-institutional collaborations allowing for the inclusion of such rare entities in clinical trials would be the ideal starting point to fully understand the biological behavior, response to treatment, and best treatment sequence of VHs in the era of personalized medicine.

## Figures and Tables

**Table 1:** Oncological outcomes of MIBC treated with RC +/− NAC and/or AC.

Author (Year)	Cohort (n)	Design	HS (%)	Treatment	Oncological Outcomes—Main Findings
Martini et al. (2021) [18]	2422	Retrospective	Non-urothelial (9%); mixed variants (10%); squamous diff. (29%); micropapillary (14%); other (29%)	NAC 10%, AC 17%, NAC + AC 1%	**5 yr OS variants**: 48% **5 yr CSS variants**: 62% **10-yr RFS**: Pure UC: 51% VHs: 30%
Claps et al. (2023) [19]	1082	Retrospective	Pure UC 72.5% HSs 27.5%: Squamous diff. (15.3%) Micropapillary (3.7%) Sarcomatoid (2.7%) Glandular (1.7%) Lymphoepithelioma-like (1.3%) Small-cell (1.2%) Clear-cell (0.7%) Nested (0.6%) Plasmacytoid (0.3%)	RC	**DSS at Univariable analysis** Sarcomatoid HR 1.61, (95% CI 1.01–2.59), *p* = 0.047 Plasmacytoid HR 6.21, (95% CI 1.99–19.4), *p* = 0.002 Small-cell HR 1.93, (95% CI 1.09–3.73), *p* = 0.03 Lymphoepithelioma-like HR 0.20, (95% CI 0.05–0.82), *p* = 0.025 **DSS at Multivariable analysis** Plasmacytoid HR 3.37 (95% CI 1.06–10.7), *p* = 0.04 Small-cell HR 1.93 (95% CI 1.02–3.79), *p* = 0.04
Naspro et al. (2021) [20]	525	Retrospective	Pure UC (75%) Squamous diff. (14.9%) Glandular diff. (1.5%) Nested (1.5%) Micropapillary (2.9%) Small Cell (1.3%) Sarcomatoid (1.7%) Plasm-Troph diff.-MC (1.2%)	RC	**Recurrence** Pure UC Ref. Squamous HR 1.0 (95% CI 0.67–1.47), *p* = 0.9 Glandular HR 0.9 (95% CI 0.28–2.79), *p* = 0.8 Nested HR 0.7 (95% CI 0.2–2.40), *p* = 0.6 Micropapillary HR 0.8 (95% CI 0.33–1.73), *p* = 0.5 Small Cell HR 1.1 (95% CI 95% CI 0.42–2.72), *p* = 0.9 Sarcomatoid HR 3.2 (CI 95% 1.40–7.06), *p* = 0.005 Plasm-Troph-MC HR 2.3 (95% CI 0.56–9.61), *p* = 0.2 **CSM** Pure UC Ref. Squamous HR 1.7 (95% CI 1.12–2.56), *p* = 0.01 Glandular HR 3.2 (95% CI 0.41–24.86), *p* = 0.3 Nested HR 1.7 (95% CI 0.51–5.53), *p* = 0.4 Micropapillary HR 1.4 (95% CI 0.61–3.44), *p* = 0.4 Small Cell HR 3.8 (95% CI 1.39–10.26), *p* = 0.009 Sarcomatoid HR 11.4 (95% CI 4.49–28.74), *p* < 0.001 Plasm-Troph-MC HR 6.2 (95% CI 1.43–26.47), *p* = 0.01 **OM** Pure UC Ref. Squamous HR 1.0 (95% CI 0.68–1.40), *p* = 0.9 Glandular HR 0.2 (95% CI 0.02–1.11), *p* = 0.06 Nested HR 0.9 (95% CI 0.28–2.90), *p* = 0.9 Micropapillary HR 0.9 (95% CI 0.45–1.81), *p* = 0.8 Small-cell HR 1.4 (95% CI 0.55–3.58) *p* = 0.5 Sarcomatoid HR 2.3 (95% CI 0.99–5.41), *p* = 0.06 Plasm-Troph-MC HR 1.6 (95% CI 0.40–6.66), *p* = 0.5
Janopaul-Naylor et al. (2021) [21]	2093	Retrospective	Squamous Cell carcinoma (35%) Adenocarcinoma (15%) Neuroendocrine (15%) Micropapillary (10%) Other (25%)	RC	**Median OS**: 19.1 months **5 yr OS**: 33.2%
Moschini et al. (2017) [22]	1067	Retrospective	Pure UC (68.3%) Sarcomatoid (2%) Lymphoepitelial (0.9%) Small cell (1.8%) Squamous diff. (10.2%) Micropapillary (8.3%) Glandular diff. (2.2%) Mixed variant (3.2%) Other variant (3.1%)	RC	**OM** Pure UC: Ref. Sarcomatoid HR 1.14 (95% CI 0.55–2.23), *p* = 0.7 Lymphoepitelial HR 1.24 (95% CI 0.63–1.83), *p* = 0.9 Small-cell HR 2.97 (95% CI 1.47–6.00), *p* = 0.002 Squamous HR 1.29 (95% CI 0.91–1.82), *p* = 0.1 Micropapillary HR 0.80 (95% CI 0.47–1.36), *p* = 0.4 Glandular HR 1.25 (95% CI 0.45–3.47), *p* = 0.7 Mixed variants HR 1.40 (95% CI 0.79–1.89), *p* = 0.2 Other variants HR 1.18 (95% CI 0.73–1.89), *p* = 0.5 **CSM** Pure UC: Ref. Sarcomatoid HR 1.18 (95% CI 0.48–2.91), *p* = 0.8 Lymphoepitelial HR 1.32 (95% CI 0.78–2.15), *p* = 0.9 Small-cell HR 3.30 (95% CI 1.56–7.02), *p* = 0.003 Squamous HR 1.29 (95% CI 0.87–1.92), *p* = 0.6 Micropapillary HR 0.66 (95% CI 0.35–1.26), *p* = 0.3 Glandular HR 1.54 (95% CI 0.55–4.34), *p* = 0.8 Mixed variants HR 1.36 (95% CI 0.71–2.61), *p* = 0.3 Other variants HR 1.41 (95% CI 0.87–2.28), *p* = 0.3
Stroman et al. (2019) [23]	430	Retrospective	Pure UC (83%) All variants (17%): Squamous diff. (9.5%) Glandular diff. (3%) Micropapillary (1.8%) Plasmocytoid(0.69%) Nested (0.9%) Microcystic (0.9%) Sarcomatoid (2.0%) Clear cell (0.2%)	RC	**2 yr OS:** pure UC 85%; all variants 71% **5 yr OS:** pure UC 70%; all variants 47% **2 yr CSS:** pure UC 87%; all variants 72% **5 yr CSS:** pure UC 75%; all variants 54%
Monn et al. (2015) [24]	624	Retrospective	Pure UC (74%) Squamous diff. (10.9%) Micropapillary (4.5%) Plasmacytoid (4%) Sarcomatoid (2.4%) Other (4.2%)	RC +/− AC	**Median OS (months):** Pure UC NR Squamous diff. 68 Micropapillary 24 Plasmocytoid 22 Sarcomatoid NR Other NR
Fairey et al. (2012) [25]	1380	Retrospective	Pure UC (97.6%) Micropapillary (2.4%)	RC +/− NAC +/− AC	**5 yr OS**: Pure UC 59% Micropapillary 67% **5 yr RFS**: Pure UC 67% Micropapillary 58%

Abbreviations are as follows: MIBC: muscle invasive bladder cancer; RC: radical cystectomy; OS: overall survival; DSS: disease-specific survival; CSS: cancer-specific survival; NAC: neoadjuvant chemotherapy; AC: adjuvant chemotherapy; UC: urothelial carcinoma; VH: variant histology; HR: hazard ratio; CI: confidence interval, HS: histological subtype; OM: overall mortality; NR: not reported; Plasm-Troph-MC: Plasmocytoid, trophoblastic differentiation and microcystic subtype; Mixed variants: presence of 2 or more variants on the same specimen.

**Table 2:** Oncological outcomes of MIBC treated with TMT.

Author (Year)	Cohort (n)	Design	HS (%)	Treatment	Oncological Outcomes—Main Findings
Janopaul-Naylor et al. (2021) [21]	356	Retrospective	Squamous cell carcinoma (26%) Adenocarcinoma (11%) Neuroendocrine diff.(38%) Micropapillary (5%) Other (21%)	Maximal TURBT, chemotherapy, and radiotherapy	**Median OS**: 19 months **5 yr OS**: 26.4%
Krasnow et al. (2016) [26]	303	Retrospective	Pure UC (78.2%) VUC (21.8%): Squamous diff. (7.9%) Glandular diff. (7.6%) Squamous and glandular diff. (0.7%) Sarcomatoid (2.7%) Micropapillary (1%) Neuroendocrine (1%) Nested (0.3%) Nested and glandular (0.3%) Clear cell (0.3%)	Maximal TURBT, chemotherapy and radiotherapy	**5 yr OS**: PUC 61%; VUC 52% **10 yr OS**: PUC 42%; VUC 42% **5 yr DSS**: PUC 75%; VUC 64% **10 yr DSS**: PUC 67%; VUC 64%
Van de Kamp et al. (2018) [27]	65	Retrospective	Small-cell carcinoma (100%)	Maximal TURBT, chemotherapy and radiotherapy	**Median OS**: 52 months **Median CSS**: 134 months **Median RFS**: 22 months
Nagumo et al. (2020) [28]	148	Retrospective	Pure UC (92.6%) Variants (7.4%) of which: Divergent diff., (squamous, glandular) (64%) sarcomatoid, plasmacytoid, signet ring cell, and clear cell (36%)	Maximal TURBT, chemotherapy and radiotherapy	**3 yr OS**: PUC 87%; Variants 88% **5 yr OS**: PUC 84%; Variants 75% **3 yr CSS**: PUC 90%; Variants 88% **5 yr CSS**: PUC 89%; Variants 75% **3 yr PFS**: PUC 84%; Variants 81% **5 yr PFS**: PUC 80%; Variants 58%
Barletta et al. (2022) [29]	3846	Retrospective	Pure UC (94.3%) Neuroendocrine Carcinoma (2.7%) Squamous cell carcinoma (2.2%) Adenocarcinoma (0.8%)	TURBT, chemotherapy, radiotherapy	3 yrs CSM free-survival: 57% (pure UC), 51% (neuroendocrine), 35% (squamous), and 60% (adenocarcinoma)

Abbreviations are as follows: MIBC: muscle invasive bladder cancer; OS: overall survival; DSS: disease-specific survival; CSS: cancer-specific survival; UC: urothelial carcinoma; HS: histological subtypes; VUC: variant urothelial carcinoma; PFS: progression-free survival; TURBT: transurethral resection bladder tumor.

## Data Availability

Not applicable.
